# L1CAM-Enriched Extracellular Vesicle MicroRNAs in Mild Cognitive Impairment and Alzheimer’s Disease: An Exploratory Study

**DOI:** 10.3390/ijms27209301

**Published:** 2026-10-20

**Authors:** Selene De Benedittis, Patrizia Spadafora, Beatrice Maria Greco, Armando Di Palo, Olivier Gallo, Domenico Nuzzo, Pasquale Picone, Elvira Brunelli, Settimio Sesti, Alberto Montesanto, Giuseppe Passarino, Katia Risoleo, Francesca Ferrise, Rosanna Colao, Raffaele Maletta, Giovanna Marchese, Francesca Cavalcanti

**Affiliations:** 1 Institute for Biomedical Research and Innovation (IRIB), Italian National Research Council (CNR), Loc. Burga 44, 87050 Mangone, Italy; selene.db90@gmail.com (S.D.B.); patrizia.spadafora@cnr.it (P.S.); beatricemariagreco@gmail.com (B.M.G.); olivier.gallo@cnr.it (O.G.); 2 Department of Biology, Ecology and Earth Sciences (DiBEST) University of Calabria, 87036 Rende, Italy; elvira.brunelli@unical.it (E.B.); settimio.sesti@unical.it (S.S.); alberto.montesanto@unical.it (A.M.); giuseppe.passarino@unical.it (G.P.); 3 Genomix4Life Srl, 84081 Baronissi, Italy; armando.dipalo@genomix4life.com (A.D.P.); giovanna.marchese@genomix4life.com (G.M.); 4 Institute for Biomedical Research and Innovation (IRIB), Italian National Research Council (CNR), Via U. La Malfa 153, 90146 Palermo, Italy; domenico.nuzzo@cnr.it (D.N.); pasquale.picone@cnr.it (P.P.); 5 Regional Neurogenetic Centre (CRN), Department of Primary Care, ASP Catanzaro, 88046 Lamezia Terme, Italy; katiarisoleo@gmail.com (K.R.); francescaferrise@libero.it (F.F.); ros.colao@gmail.com (R.C.); malettar@yahoo.it (R.M.)

**Keywords:** Alzheimer’s disease, dementia, aging, microRNAs, biomarkers, L1CAM-enriched EVs

## Abstract

Early diagnosis of Alzheimer’s disease (AD) and the identification of reliable molecular biomarkers remain critical clinical needs. In this context, extracellular vesicles (EVs) enriched by L1CAM immunocapture—L1CAM-enriched EVs—a fraction widely used to obtain material of putative neuronal origin, have been proposed as a minimally invasive source of information on brain molecular changes. We conducted next-generation sequencing (NGS) of microRNAs (miRNAs) from L1CAM-enriched EVs isolated from the plasma of 11 AD patients, 11 mild cognitive impairment (MCI) individuals, and 14 healthy controls (CTRs) to identify a candidate molecular signature. Our analysis identified five differentially expressed miRNAs—miR-423-5p, miR-122-5p, miR-151b, miR-16-5p, and miR-149-3p—in MCI individuals. Three of these—miR-423-5p, miR-122-5p and miR-151b—were higher in MCI and lower in AD, a pattern that differentiated the two clinically defined groups. Furthermore, enrichment analysis of the predicted targets of the differentially expressed miRNAs identified processes relevant to neurodegeneration, including synaptic organisation, cytoskeletal dynamics, and protein degradation. Overall, these preliminary findings identify blood-based L1CAM-enriched EV miRNAs as candidates for further evaluation in cognitive impairment. While validation in larger, biomarker-confirmed and independent cohorts is required, this exploratory study provides data that may guide the design of future biomarker studies.

## 1. Introduction

Dementia is the leading cause of senile disability worldwide, currently affecting more than 50 million individuals, an estimate expected to triple by 2050, posing a significant challenge to global health [1]. Alzheimer’s disease (AD) is the most prevalent form of dementia characterized by gradual memory loss and impairment of various cognitive functions [2]. Although the aetiology of the disease remains elusive, the accumulation within the brain of amyloid-beta (Aβ) plaques and neurofibrillary tangles (NFTs), as well as neuronal and synaptic loss, are the disease’s pathological features, leading to cognitive decline in a progressive manner [3,4].

To date, despite the remarkable diagnostic accuracy and advancements in brain imaging techniques such as positron emission tomography (PET) and cerebrospinal fluid (CSF) biomarkers, the high costs and restricted accessibility of PET, along with the invasive nature of CSF sampling, confine their application to specialized centres and hamper broad screening efforts [5]. Further, AD is often diagnosed at a relatively advanced stage [6] and, by this point, irreversible neuronal damage has already occurred.

The pathological alterations that occur in the brain leading to AD begin in the preclinical stage, approximately 20 years prior to the onset of clinical symptoms. As pathology advances, individuals progress into a temporary state of mild cognitive impairment (MCI), which is considered a prodromal stage of AD. MCI is characterized by an objective cognitive deficit that, however, is not severe enough to substantially disrupt normal daily activities [7]. Although certain patients with MCI remain stable or revert to normal cognition, there exists a considerable risk of progression to AD, with an estimated incidence rate of 10–15% [8].

The long preclinical period and the MCI phase highlight how crucial it is to find early biomarkers to provide timely intervention meant to delay the disease progression. In addition, there is an urgent demand for less expensive and invasive AD biomarkers capable of replacing or improving existing ones.

Extracellular vesicles (EVs) are a heterogeneous family of membrane-bound particles ranging from nanoscale to microscale in size released by several cell types into extracellular space [9]. They have gained considerable attention as potential biomarkers for central nervous system diseases owing to their ability to cross the blood–brain barrier and transport a molecular cargo, including nucleic acids, lipids, and proteins, that mirrors the biological state of their parental cells [10,11,12,13]. Therefore, molecular cargo analyses of brain-derived EVs isolated from systemic biofluids may offer a minimally invasive window into the brain.

In recent years, it has been demonstrated that miRNAs play an important role in neurodegeneration. These small non-coding RNA molecules are important regulators of gene expression, acting mainly at the post-translational level. Furthermore, around 70% of known miRNAs are expressed in the brain, and several studies have emphasized their probable involvement in neurodegenerative diseases [14].

EVs are essential carriers of miRNAs, however, in body fluids, miRNAs are also bound to proteins such as those of the Argonaute family. Therefore, analyses of circulating miRNAs lack clear information about their cellular origin, and identifying their cellular origin remains a challenge. In this context, enrichment strategies targeting EV subpopulations of putative neuronal origin, such as L1CAM immunocapture, have been proposed as a way to reduce the contribution of the systemic EV background and to obtain miRNA profiles more informative of the CNS compartment.

While several studies have suggested that miRNAs from EVs of putative neuronal origin may serve as valuable diagnostic biomarkers for AD, very few studies have explored their expression by next-generation sequencing (NGS) [15,16]. Unlike targeted approaches, NGS would provide an unbiased, comprehensive landscape of the miRNA content of the L1CAM-enriched EV fraction in both AD and MCI individuals.

In this exploratory study, we performed a high-throughput sequencing analysis of miRNAs from L1CAM-enriched EVs isolated from the plasma of patients with AD, individuals with MCI, and healthy controls (CTR). The inclusion of an MCI group allowed us to characterise this fraction in individuals with mild cognitive impairment as well as in established dementia, with the aim of identifying candidate molecular differences for evaluation in future studies.

## 2. Results

### 2.1. Characterization of L1CAM-Enriched EVs

The L1CAM-enriched EV fraction was characterized using a single pooled preparation. NTA, through to particle-by-particle analysis, allows the accurate resolution of heterogeneous mixtures of vesicles that differ in size. The pooled preparation showed a population of particles with a mean size ± SD = 142 ± 66 nm (mode = 107), consistent with small EVs, and an approximate particle concentration of 4.67 × 10^11^ particles/mL (Figure S1A). Further, TEM analysis showed the presence of heterogeneous vesicles with the expected size and morphology (Figure S1B). Additionally, the presence of the EV-associated markers CD63 and ALIX and the multiple neuronally enriched markers L1CAM and β-III-tubulin [17] (Figure S1C), was confirmed through Western blotting. Together, these findings confirm that the immunocaptured fraction contains small EVs displaying L1CAM and β-III-tubulin.

### 2.2. miRNA Profiling of L1CAM-Enriched EVs

Small RNA expression profiling was performed by next-generation sequencing on L1CAM-enriched EVs samples from individuals with AD, MCI, and healthy controls. After adapter trimming and quality filtering, only high-quality reads were retained and aligned to the human reference genome. Principal component analysis (PCA) did not reveal a clear separation among the three groups (Figure S2). This unsupervised analysis should only be understood descriptively and does not suggest any diagnostic categorization or predictive efficacy. Across all samples, a total of 120 unique miRNAs were initially detected.

### 2.3. Differentially Expressed miRNAs in L1CAM-Enriched EV

**Figure 1:**
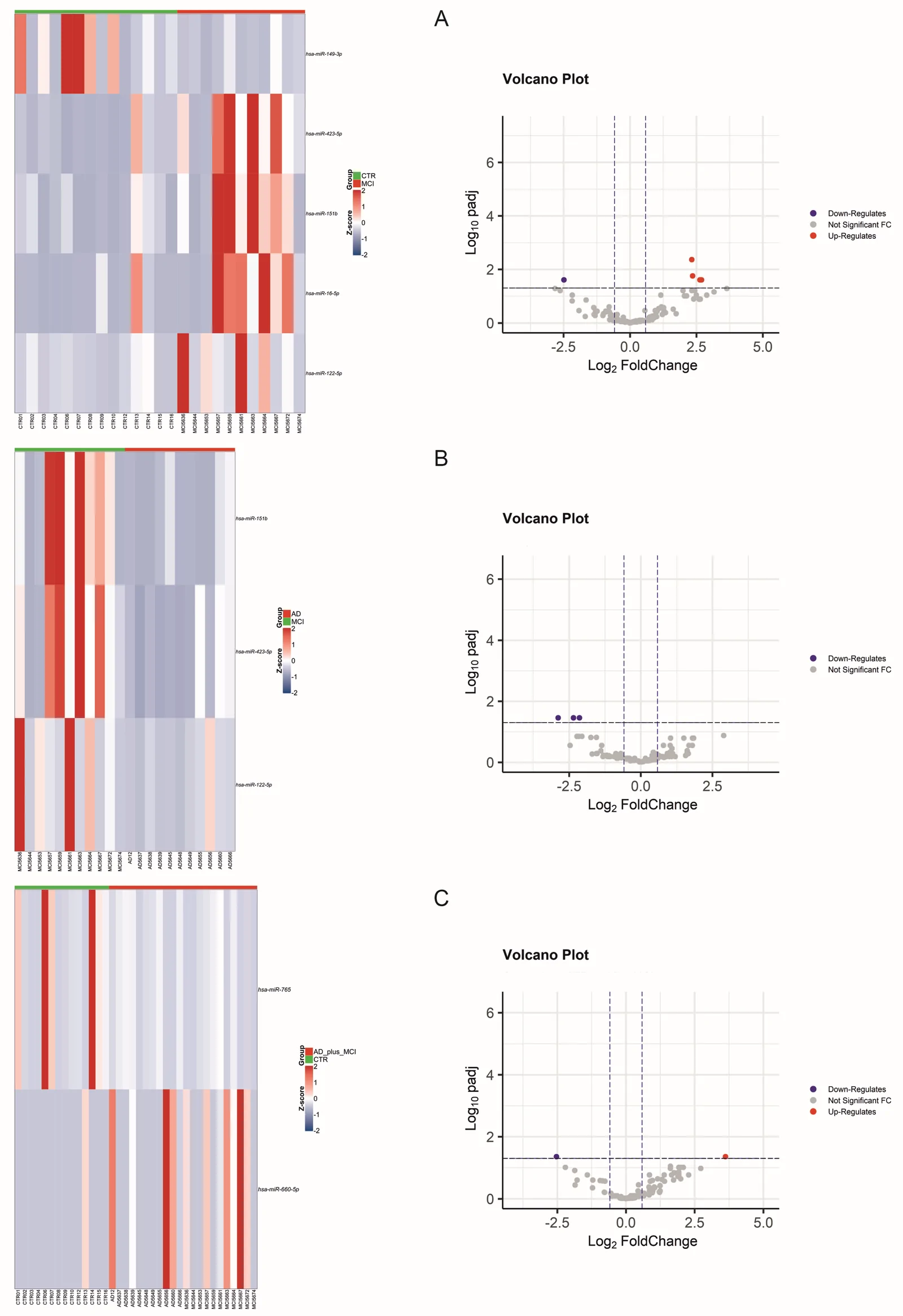
miRNA profiling analysis across the study groups. (**A**) MCI vs. CTR; (**B**) AD vs. MCI; (**C**) AD + MCI vs. CTR. For each comparison, the left panel shows the heatmap of supervised hierarchical clustering analysis based on the expression profiles of differentially expressed miRNAs (DEmiRNAs) identified using the thresholds *p*adj ≤ 0.05 and FC ≥ 1.50 or ≤−1.50). Expression values lower or higher than the median are represented in blue and red, respectively. The right panels display the corresponding volcano plots of DEmiRNAs (*p*adj ≤ 0.05 and FC ≥ 1.50 or ≤−1.50), where significantly upregulated miRNAs are shown in red, significantly downregulated miRNAs in blue, and non-significant miRNAs in grey. Dashed lines indicate the fold-change and adjusted *p*-value significance thresholds applied for differential expression analysis.

Differential expression analysis between MCI individuals and healthy controls identified five significantly dysregulated miRNAs (FDR ≤ 0.05). Four miRNAs were upregulated in MCI—miR-423-5p (FC = 5.007; FDR = 0.004), miR-122-5p (FC = 5.122; FDR = 0.018), miR-151b (FC = 6.200; FDR = 0.025), and miR-16-5p (FC = 6.459; FDR = 0.025)—while miR-149-3p was significantly downregulated (FC = −5.611; FDR = 0.025). Applying a more stringent FC threshold of ≥1.50 or ≤−1.50 four miRNAs remained upregulated and one remained downregulated (Table 1 and Table S1, Figure 1A).

When comparing AD and MCI cohorts, three miRNAs were significantly differentially expressed (FDR ≤ 0.05), all showing reduced expression in AD relative to MCI: miR-423-5p (FC = −4.40), miR-122-5p (FC = −5.09), and miR-151b (FC = −7.37) (Table 1 and Table S2, Figure 1B). These miRNAs had shown higher levels in MCI than in CTR, so their levels differed in opposite directions in the two patient groups. The combined analysis of MCI and AD patients versus healthy controls identified two significantly dysregulated miRNAs: hsa-miR-660-5p (FC = 12.309; FDR = 0.044) and hsa-miR-765 (FC = −5.779; FDR = 0.044), which differed between patients and controls irrespective of clinical stage (Table 1 and Table S3, Figure 1C). Conversely, miR-423-5p, miR-122-5p, and miR-151b did not reach significance in this combined comparison (Table 1), consistent with their opposite directions of difference in the two patient groups, which attenuate one another when MCI and AD are analysed together. The combined analysis therefore identifies differences shared across clinical stages and does not summarise the stage-specific results.

In contrast, the direct comparison between AD patients and healthy controls did not identify any miRNA surpassing the FDR-adjusted significance threshold of 0.05 (Table S4).

**Figure 2:**
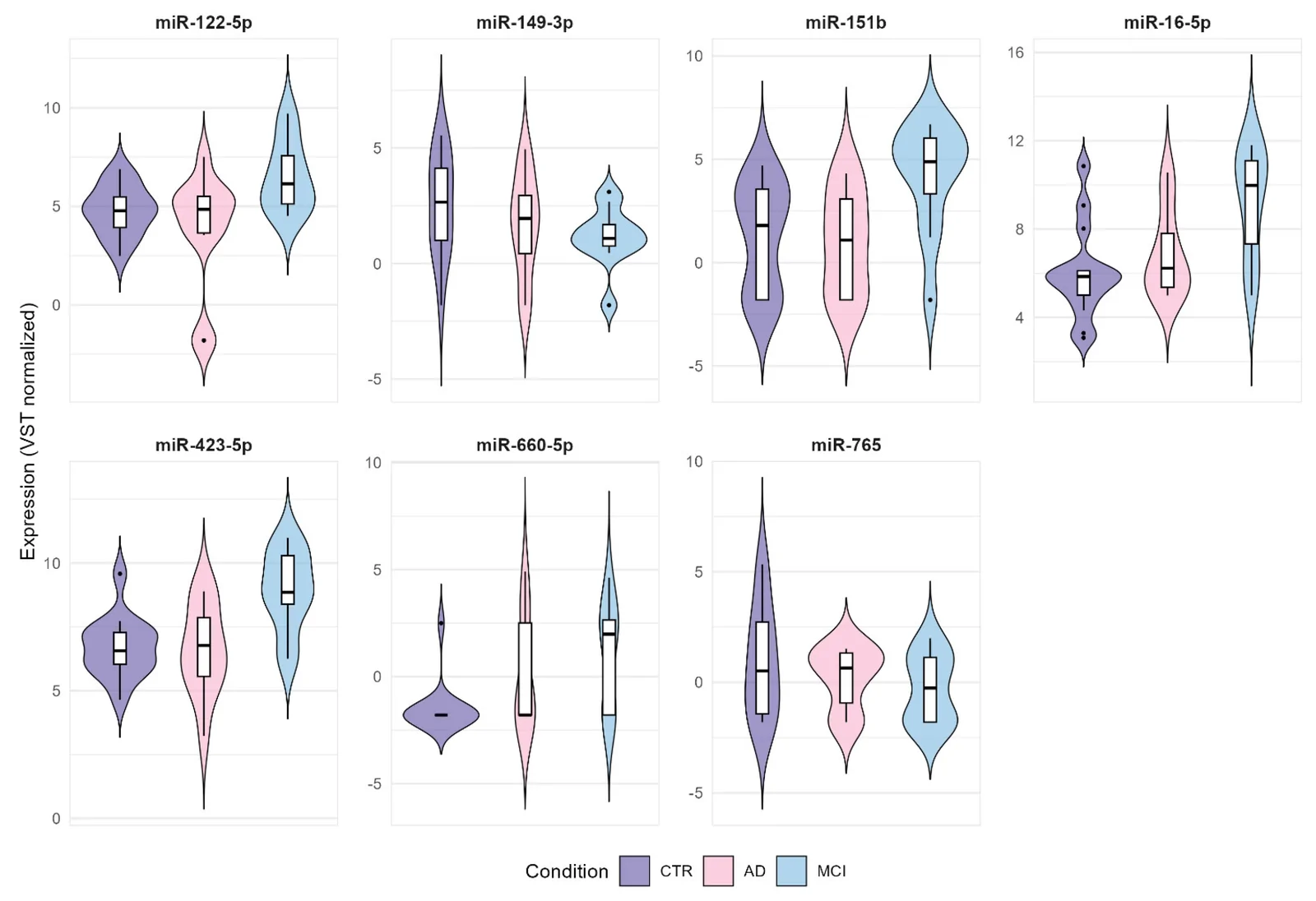
Violin plots showing the miRNAs expression in healthy controls (CTR, purple), patients with Alzheimer’s disease (AD, pink), and individuals with mild cognitive impairment (MCI, light blue). miRNA expression levels were normalized using the variance stabilization.

The differences detected in this dataset were therefore more pronounced in the MCI group than in the AD group. Taken together, the opposite directions of change observed for miR-423-5p, miR-122-5p, and miR-151b in the MCI and AD groups distinguish these two clinically defined populations; whether this pattern reflects a change over time within individuals cannot be established from a cross-sectional comparison, and validation in longitudinal, biomarker-confirmed cohorts is required. The expression trends of statistically significant miRNAs across the CTR, MCI, and AD cohorts are evident in the violin plots (Figure 2).

### 2.4. Enrichment Analysis of Predicted Targets

Functional enrichment analysis of the predicted targets of the differentially expressed miRNAs identified biological processes significantly enriched across the different comparisons (MCI vs. CTR, AD vs. MCI, and AD + MCI vs. CTR). Gene Ontology (GO) analysis, focused on the Biological Process (BP) category, identified pathways related to cellular organization, signal transduction, protein catabolism, and neuronal development among the most significantly enriched terms (FDR < 0.05).

**Figure 3:**
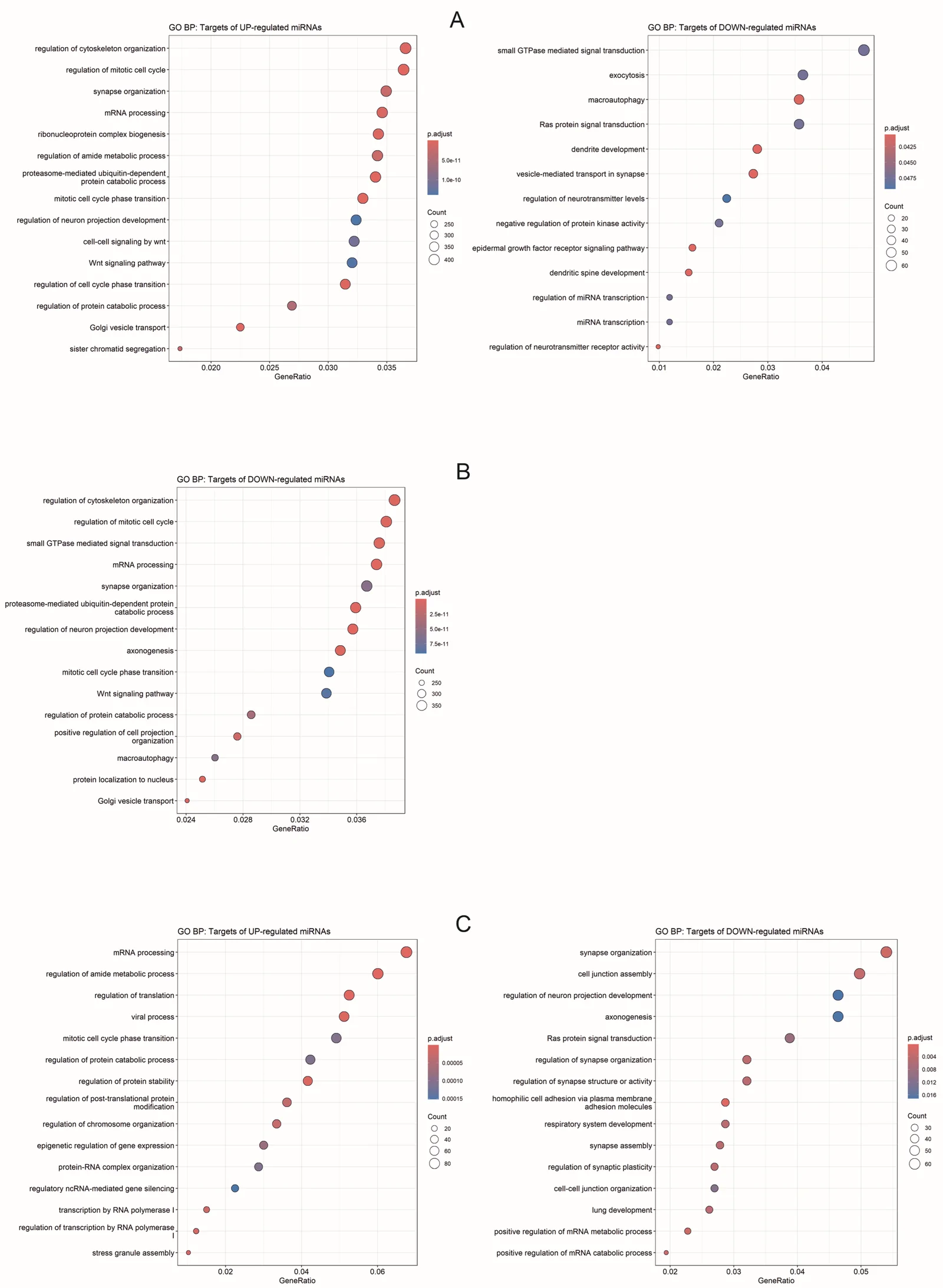
Gene Ontology Biological Process (GO-BP) enrichment analysis of predicted target genes associated with differentially expressed miRNAs. (**A**) MCI vs. CTR; (**B**) AD vs. MCI; (**C**) AD + MCI vs. CTR. For each comparison, the left panel shows the GO-BP enrichment analysis of target genes associated with upregulated miRNAs, whereas the right panel displays the GO-BP enrichment analysis of target genes associated with downregulated miRNAs. Dot plots were generated according to GeneRatio, adjusted *p*-value (*p*.adjust), and number of enriched genes (Count). Bubble size represents the number of genes involved in each biological process, while colour intensity reflects the enrichment significance based on adjusted *p*-values.

In the comparison between and MCI individuals and healthy controls, 342 biological processes were identified in the targets of upregulated miRNAs and 13 in the targets of downregulated miRNAs (Table S5). The targets of upregulated miRNAs were associated with processes related to cytoskeleton and synapse organization (regulation of cytoskeleton organization, synapse organization), as well as RNA regulatory mechanisms (mRNA processing, ribonucleoprotein complex biogenesis) and protein degradation (proteasome-mediated protein catabolic process). Enrichment of the Wnt signalling pathway was also observed, suggesting a role in cell–cell communication and neuronal maintenance processes. Conversely, the targets of downregulated miRNAs showed enrichment in intracellular signalling pathways (small GTPase-mediated signal transduction, Ras signalling), neurotransmission (regulation of neurotransmitter levels, vesicle-mediated transport in synapse, exocytosis), and neuronal development (dendrite development and dendritic spine development). Processes related to intracellular degradation (macroautophagy) and regulation of miRNA transcription were also represented. Overall, the predicted targets of the miRNAs differentially expressed in MCI were enriched for processes related to neuronal signalling mechanisms, synaptic transmission and gene expression regulatory processes, suggesting a possible involvement of both synaptic function and proteostasis at this clinical stage (Figure 3A).

In the comparison between patients with AD and those with MCI, the analysis identified 351 enriched biological processes in the targets of downregulated miRNAs (Table S6). The most represented terms included regulation of cytoskeleton organization and small GTPase-mediated signal transduction, suggesting involvement in the regulation of cellular structure and cytoskeletal dynamics. Synaptic and neuronal processes were also enriched (synapse organization, axonogenesis), together with mechanisms related to cell cycle control (regulation of mitotic cell cycle). Particularly noteworthy is the enrichment of processes associated with protein degradation and intracellular turnover, including proteasome-mediated ubiquitin-dependent protein catabolic process, regulation of protein catabolic process, and macroautophagy, which together concern proteostasis. Processes related to intracellular trafficking (Golgi vesicle transport) and protein localization were also involved. Overall, the predicted targets of the miRNAs differentially expressed between the MCI and AD groups were enriched for processes related to synaptic dysfunction, structural alterations, and impairment of protein degradation, pointing to a possible involvement of these functions in the differences observed between the two groups (Figure 3B).

In the overall comparison between CTR and patients (MCI + AD), the analysis identified 145 enriched BP among the targets of upregulated miRNAs and 33 among the targets of downregulated miRNAs (Table S7). Targets of upregulated miRNAs were mainly associated with processes related to gene expression regulation, including mRNA processing, regulation of translation, and epigenetic regulation of gene expression, indicating involvement in both transcriptional and post-transcriptional control mechanisms. Enriched terms also included processes related to protein stability and metabolism (regulation of protein stability, regulation of amide metabolic process), macromolecular complex organization (protein–RNA complex organization), and dynamic cellular processes such as cell cycle progression and stress response (mitotic cell cycle phase transition, stress granule assembly). Overall, these findings concern the regulation of gene expression and protein synthesis. In contrast, targets of downregulated miRNAs showed a strong enrichment in neuronal and synaptic processes, including synapse organization, synapse assembly, and regulation of synaptic plasticity, as well as neuronal development processes such as axonogenesis and regulation of neuron projection development. Processes related to cell adhesion and junction organization (cell junction assembly, cell–cell junction organization) were also represented, along with intracellular signalling pathways (Ras protein signal transduction). Collectively, the predicted targets of the miRNAs differentially expressed between patients and controls were enriched for processes related to synaptic plasticity and neuronal connectivity, suggesting a possible involvement of these functions in cognitive impairment (Figure 3C).

## 3. Discussion

MCI represents a critical transitional phase between normal aging and dementia, making it an optimal window for evaluating early biomarkers and timely therapeutic interventions to delay dementia onset [18]. This is particularly relevant, as AD is recognized as a continuum in which pathophysiological changes begin decades before overt clinical symptoms [19]. To detect these early neuronal shifts, identifying biomarkers that directly reflect molecular changes in the brain environment is essential.

In our exploratory study, we investigated this issue using a high-throughput methodology to characterize the miRNA landscape within L1CAM-enriched EVs, evaluating non-invasive molecular markers in the early stages of dementia. Although previous research has explored the biomarker potential of EV-miRNAs in MCI and AD, most studies relied on total EVs from plasma or serum [20]. The use of L1CAM immunocapture was intended to reduce the contribution of the systemic EV background, although, as discussed below, the extent of this reduction was not quantified in the present study. A growing body of evidence highlights the utility of miRNAs from EVs of putative neuronal origin in tracking disease trajectory. Recently, Reho and colleagues, using EVs immunocaptured with NLGN3 and GAP43, identified a miRNA signature associated with increased AD risk in preclinical individuals who progressed to clinical AD in less than 20 years, highlighting more significant transcriptional changes in preclinical AD than during the symptomatic phase [15]. Previously, Kumar et al. analysed a panel of eight miRNAs shuttled in different subtypes of brain cell-derived EVs, including L1CAM-enriched EVs. They identified miRNAs distinguishing, with high diagnostic accuracy, MCI, MCI progressing to Alzheimer’s (MCI-AD), and AD dementia, correlating these molecular changes with temporal cortical thinning [16].

Consistently, we identified a candidate signature of five miRNAs in L1CAM-enriched EVs—mir-423-5p, mir-122-5p, mir-151b, mir-16-5p, and mir-149-3p—differentially expressed in MCI individuals, which may represent markers of early stages of neurodegeneration. To our knowledge, this is the first untargeted NGS-based profiling of miRNAs in L1CAM-enriched EV preparations in MCI, extending previously reported targeted panels.

Interestingly, the differences in miR-423-5p, miR-122-5p, and miR-151b remained statistically significant in the comparison between the MCI and AD groups, with higher levels in MCI and lower levels in AD. Because the two groups were assessed cross-sectionally and the MCI participants were not characterised by amyloid or tau biomarkers, this pattern cannot be interpreted as a change occurring within individuals as the disease advances; it describes a difference between two clinically defined populations. The two groups also differed considerably in clinical severity, with a mean MMSE of 20.5 in the MCI group and 12.8 in the AD group, the latter indicating moderate to severe dementia. The comparison therefore spans a substantial clinical distance rather than adjacent stages, which may contribute to the magnitude of the differences observed. One possible interpretation of the reciprocal expression pattern of these three miRNAs, which our data cannot test, is that it reflects a stage-dependent difference in the regulatory state of the cells of origin, with higher levels in the MCI group representing a compensatory or stress-related response and lower levels in established AD reflecting a loss of that response or of cellular integrity. Such a pattern might also account for the absence of significant differences between AD patients and controls. This interpretation remains speculative: testing it would require longitudinal sampling in biomarker-confirmed cohorts, together with functional studies.

The combined analysis of MCI and AD patients versus controls identified two further miRNAs, miR-660-5p and miR-765, which differed between patients and controls irrespective of clinical stage. Neither reached significance in the individual comparisons, so the combined result may reflect a difference of smaller magnitude detectable only with the larger group size or may arise from pooling two clinically distinct populations. These possibilities cannot be distinguished from the present data, and this finding should be regarded as provisional pending replication.

The biological relevance of the differentially expressed miRNAs identified here can be considered in the light of existing literature, which links several of them to pathological hallmarks, although these mechanisms were established in other experimental systems and their relevance to the present findings remains hypothetical. A recent meta-analysis identified miR-149-3p as a central node in the mRNA-miRNA interaction network, indicating a key role as a regulator of early neurodegenerative processes [21]. Consistent with our analysis, the serum level of its precursor was found to be downregulated in AD patients and positively correlated with the MMSE score [22]. In other experimental systems, both miR-149-3p and miR-16-5p have been reported to target BACE1 [22,23], an enzyme known to promote Aβ accumulation by enhancing the cleavage of amyloid precursor protein (APP), a mechanism through which these miRNAs could contribute to amyloidogenic processing. Whether this applies in the context studied here was not investigated.

Beyond protein aggregation, cognitive decline and metabolic dysfunction share common pathophysiological mechanisms. In the brain, insulin resistance (IR) alters glucose metabolism, impairing synaptic function and neuronal survival, and has been implicated in AD pathogenesis [24]. A bioinformatics study by Ahmed and colleagues identified a subset of miRNAs, including miR-16-5p, as candidates linking IR to AD-related genes [25]. No data on metabolic status were available for our participants, so any relationship between miR-16-5p levels and insulin resistance in this cohort remains untested. Furthermore, Visconte et al. identified a four-miRNA signature, including miR-16-5p, specifically increased in MCI due to AD individuals [26]. Finally, FDG-PET of the brain in MCI individuals established a direct correlation between miR-16-5p levels and brain hypometabolism [27], reinforcing the potential of miR-16 to reflect early AD-related changes.

Similarly, miR-423-5p has been linked to reduced hippocampal volume, worse cognition, and higher brain amyloid deposition [28]. Evidence suggests its possible contribution to autophagy-related processes [29], which are essential in removing damaged proteins and sustaining neuronal health. Further, miR-423-5p has been shown to inhibit the translation of COX6A2, a subunit of respiratory chain complex IV [30], whether this is relevant in the present context remains hypothetical.

Regarding miR-122-5p, Subasinghe et al. reported downregulation in a cohort of cognitively impaired individuals analysed as a single group, comprising both AD and MCI patients [31], whereas we observed significant upregulation in MCI. Since both studies examined L1CAM-enriched EVs, this discrepancy is unlikely to reflect the fraction analysed. It may instead depend on cohort composition.

The role of miR-151b in AD remains to be fully elucidated. Integrated transcriptomic analysis included miR-151b in a set of miRNAs interacting with genes differentially expressed in MCI [32]. The research has identified a potential interaction between miR-151b and GPER1, a membrane-bound oestrogen receptor involved in synaptic plasticity and memory enhancement in the hippocampus [32,33]. miR-151a-5p and miR-151b have also been proposed as molecular correlates of psychiatric symptoms [34], affecting from 35 to 85% of individuals with MCI [35]. In particular, a study by Qiu and colleagues indicated a positive correlation between miR-151b and anxiety [34].

Our enrichment analyses relate the predicted targets of the differentially expressed miRNAs detected in L1CAM enriched EVs to processes previously implicated in AD pathology. In the MCI group, the BPs associated with these miRNAs concern functions relevant to neuronal homeostasis. Of particular interest is the upregulation of miRNAs targeting the mitotic cell cycle. Aberrant cell cycle re-entry in post-mitotic neurons is a known early pathological event leading to neuronal death in both MCI and AD [36,37]. Thus, the enrichment of miRNAs targeting these pathways may represent a modulatory mechanism to inhibit this harmful mitotic reactivation. The enrichment of terms related to cytoskeleton organization and Golgi transport concerns functions required for neuronal structural integrity, which have been linked to axonal degeneration in early cognitive decline [38]. Conversely, the miRNAs with lower levels in this group have predicted targets in small GTPase-mediated signal transduction, vesicle-mediated transport in the synapse, and dendritic spine development, processes central to synaptic plasticity and therefore to learning and memory [39,40].

Furthermore, terms related to macroautophagy and proteasome-mediated catabolism were also enriched, processes whose disruption has been implicated in toxic protein aggregation. Overall, the processes enriched across the comparisons converge on synaptic organisation, cytoskeletal dynamics, protein degradation, and cell cycle regulation, a convergence consistent with processes described in AD pathology. These associations, however, derive from database-annotated targets and were not verified by measurement of transcript or protein levels in our samples; therefore, they identify hypotheses for mechanistic testing rather than evidence of pathway alteration.

This study provides, to our knowledge, the first untargeted NGS-based profiling of miRNAs in L1CAM-enriched plasma EV preparations in MCI. All samples were processed in parallel under an identical protocol by the same operator, with libraries prepared in a single session and sequenced on the same flow cell, so the comparisons reported are internally consistent.

Several limitations apply to the material analysed. The isolation workflow was not characterised quantitatively: bead-only and isotype-control captures were not included, the input, unbound, and depleted fractions were not compared, and contaminant markers were not assessed, so capture recovery and enrichment were not quantified. No nuclease protection assay was performed, so the sequenced miRNAs cannot be shown to be luminal rather than surface-associated or carried by ribonucleoprotein complexes and lipoproteins. The cellular origin of the vesicles is likewise not established: L1CAM is expressed outside the CNSs, and bulk immunoblotting cannot show that L1CAM and β-III-tubulin reside in the same vesicles, so we use the term L1CAM-enriched EVs operationally and treat neuronal attribution as a hypothesis.

Further limitations concern the design. Patients and controls were recruited at two centres, so recruitment source is confounded with disease status; pre-analytical procedures were identical, and PCA revealed no group-related structure, but unrecorded site-associated differences cannot be excluded. This study is cross-sectional, so group differences cannot establish within-individual change or progression between stages. The MCI group was diagnosed clinically, without amyloid or tau biomarkers, and is aetiologically heterogeneous. Comorbidities and medication use were not systematically recorded and could not be used as covariates, although several such conditions affect circulating EV and miRNA composition.

The cohort of 36 participants was assembled as a discovery screen rather than sized on the basis of a power calculation. Power is therefore limited, effect sizes crossing a significance threshold are expected to be overestimated, and correction was applied within but not across the four comparisons, increasing the probability of false positives; no measures of diagnostic performance are reported, and none should be inferred. The miRNAs identified here were detected by sequencing alone: they were not validated on an orthogonal platform such as RT-qPCR or ddPCR, nor were they examined in an independent cohort, both of which will be required before they can be considered established candidates. Functional enrichment was performed on database-annotated targets without measuring transcript or protein levels, so the processes reported are associations with predicted targets rather than evidence of pathway alteration.

Finally, glial-derived EVs were not investigated, although they contribute to CNS homeostasis and impact the structural functions and neuroinflammatory milieu associated with cognitive decline [16,26,41]. Future studies on glial-derived EVs may reveal additional key biomarkers.

This work is therefore an exploratory, hypothesis-generating screen, requiring technical validation on an orthogonal platform and confirmation in prospectively collected cohorts with biomarker-confirmed diagnoses, balanced recruitment, and the methodological controls described above.

## 4. Materials and Methods

### 4.1. Study Cohort

Peripheral blood samples from MCI (n = 11) and AD (n = 11) patients were collected at the Regional Neurogenetic Center (ASP CZ), Catanzaro. Further, 14 CTR samples matched for age and sex and without any neurological disorders were obtained from the National Research Council (CNR), Institute for Biomedical Research and Innovation (IRIB) in Mangone, Cosenza, following approval by the ethics committee. Written informed consent was obtained from all participants for blood sample collection and the use of clinical data. AD diagnosis followed guidelines established by the National Institute of Neurological and Communicative Disorders and Stroke–Alzheimer’s Disease and Related Disorders Association (NINCDS–ADRDA). Diagnosis of MCI was performed according to the guidelines of the National Institute of Aging [42]. Clinical and family histories, neurological examinations, and neuropsychological tests were assessed for each patient [43,44,45]. Diagnoses were based on clinical and neuropsychological assessments; amyloid, tau, and neurodegeneration biomarkers (CSF or PET) were not available for any participant, and the cohorts were therefore not characterised with respect to underlying aetiology. The details of the study cohorts are provided in Table 2. The cohort was not sized on the basis of a formal power calculation. This study was designed as a discovery screen intended to generate candidates for subsequent evaluation.

For all participants, irrespective of recruitment site, blood samples were collected into EDTA-containing tubes, and the first centrifugation (2500× *g* for 10 min at 4 °C) was performed within 2 h after collection; plasma was stored at −80 °C until use. After plasma storage, all samples followed an identical processing path: EV isolation, L1CAM immunocapture, storage of the immunocaptured fraction at −80 °C, RNA extraction, and storage of the extracted RNA at −80 °C. The number and sequence of freeze–thaw cycles was therefore the same for all samples.

### 4.2. Isolation of Total EVs and L1CAM Immunocapture

Total EVs were isolated from 500 µL of plasma using ExoQuick (EXOQ5TM-1; System Biosciences, Palo Alto, CA, USA). Briefly, aliquots were thawed at 4 °C and centrifuged at 3000× *g* for 15 min. Plasma was then defibrinated by mixing it with 5 µL of thrombin for 5 min at room temperature, followed by centrifugation. The resulting supernatant was incubated for 30 min at 4 °C with 126 µL of ExoQuick precipitation solution. After centrifugation, the EV pellets were resuspended in 500 µL of 0.2 µm filtered 1X PBS, along with 1X protease and phosphatase inhibitor cocktail. ExoQuick precipitation was used as a concentration step only; the resulting preparation served as input for L1CAM immunocapture, which was performed to select the L1CAM-positive EV subpopulation. All downstream analyses were performed on the immunocaptured eluate.

For L1CAM immunocapture, total EVs were mixed with 5 µg of anti-human biotinylated L1 Cell Adhesion Molecules (L1CAM) antibody, clone 5G3 (eBioscience, San Diego, CA, USA) in 40 µL of 3% bovine serum albumin (BSA). The samples were incubated on a rotator mixer for 2.5 h at 4 °C. Afterward, 15 μL of Streptavidin UltraLink Resin (Thermo Fisher Scientific, Waltham, MA, USA) in 35 μL of 3% BSA was added, and the mixtures were further incubated for 1.5 h with continuous gentle mixing. Following centrifugation, the pellets were washed with 3% BSA to eliminate unbound material. Finally, the L1CAM fraction was eluted with 200 µL of 0.1 M glycine (pH 2.5) and neutralized with 1 M Tris-HCl (pH 8.0). 1X protease and phosphatase inhibitor cocktail was added before storing the samples at −80 °C. The fraction recovered by L1CAM immunocapture is referred to in the text as “L1CAM-enriched EVs”; this term is used for operational purposes to designate the immunocaptured material, without implying a quantified enrichment factor or an established cellular origin. Specificity controls for the immunocapture step (streptavidin resin incubated without biotinylated antibody, an isotype-matched biotinylated control antibody, and analysis of the input, unbound, and L1CAM-depleted fractions) were not performed, and capture recovery and enrichment were not quantified. All samples were processed in parallel with identical reagents and the same antibody and resin lots.

### 4.3. Characterisation of L1CAM-Enriched EVs

Characterisation was performed on the final L1CAM-enriched fraction. Owing to the limited plasma volume available per participant, which was prioritised for RNA extraction and sequencing, characterisation was carried out on pooled rather than individual material: equal volumes of the L1CAM-enriched fraction from 12 randomly selected participants (CTR n = 4, MCI n = 4, AD n = 4) were combined into a single pool, on which NTA, TEM, and immunoblotting were performed. Because characterisation was performed on a single pooled preparation, inter-individual and between-group variability in particle size, concentration, and marker expression could not be assessed. Characterization of L1CAM-enriched EVs was performed using complementary methodologies, as recommended in MISEV 2023 [9]. To characterize L1CAM-enriched EVs by immunoblotting, samples were lysed using RIPA buffer 1X supplemented with 1X protease and phosphatase inhibitor cocktail. Protein concentrations of 10–20 µg from L1CAM-enriched EV lysates were subjected to separation on a 4–15% Mini-PROTEAN^®^ TGX™ precast gel (Bio-Rad, Hercules, CA, USA). Subsequently, proteins were transferred to a PVDF membrane using a semi-dry trans-blot (Bio-Rad, CA, USA). After blocking for 1 h with 3% BSA in TBS containing 0.1% Tween 20, membranes were incubated overnight at 4 °C. The following primary antibodies and corresponding dilutions were used: anti-CD63 (1:500, Invitrogen, Thermo Fisher Scientific, Waltham, MA, USA), anti-Alix (1:1000, Santa Cruz Biotechnology, Dallas, TX, USA), anti-β-III-tubulin (TUBB3, 1:500, Sigma-Aldrich, Merck, Darmstadt, Germany), and anti-L1CAM (1:500, Thermo Fisher Scientific, MA, USA). After washing with TBST, membranes were treated with HRP-conjugated, species-specific secondary antibodies for 2 h. Protein signals were detected with Western Blotting Chemiluminescence Luminol Reagent (Santa Cruz Biotechnology, TX, USA) and chemiluminescence images were obtained through X-ray film detection. The images were analysed with ImageJ software v1.54r (National Institute of Health, Bethesda, MD, USA).

For the size distribution and concentration of L1CAM-enriched EVs, nanoparticle tracking analysis (NTA) was performed using a NanoSight Pro instrument (Malvern Panalytical, Malvern, UK). Samples were diluted 1:1000 in filtered PBS; five videos were acquired. The buffer used for diluting the preparation was assessed for purity and established as a baseline, and control beads of 100 and 400 nm (Malvern, UK) were used. Captured video recordings were analysed using the NS XPLORER software version 1.1.0.6, and analysis was conducted in duplicate.

The pooled preparation was visualized using conventional transmission electron microscopy (TEM) according to the protocol of Corona et al. [46]. An amount of 4 μL of the preparation at a concentration of 10^10^ particles/mL was fixed on formvar-coated carbonated grids. After washing, the samples were stained with a uranyl acetate substitute (Electron Microscopy Sciences, Hatfield, PA, USA). Observations were performed under a JEM 1400 PLUS electron microscope (JEOL, Peabody, MA, USA).

### 4.4. RNA Isolation, Library Generation and Small RNA Sequencing

All steps up to and including RNA extraction were performed by the same operator. Total RNA from L1CAM-enriched EVs was isolated using the miRVANA Paris kit (Invitrogen, Thermo Fisher Scientific, MA, USA) according to the manufacturer’s instruction and eluted in a final volume of 60 μL. Total RNA quantification was performed using a Qubit fluorometer (Invitrogen, Thermo Fisher Scientific, Waltham, MA, USA).

Library preparation and sequencing were performed by the same external provider, Genomix4Life Srl (Baronissi, Italy). All libraries were prepared in the same experimental session and sequenced on the same flow cell. Indexed libraries were constructed using the QIAseq miRNA Library Kit (QIAGEN, Hilden, Germany), following the manufacturer’s guidelines. Library quality and concentration were evaluated using the Agilent TapeStation 4200 (Agilent Technologies, Santa Clara, CA, USA) and the Qubit fluorometer, respectively. Equimolar pooling of indexed libraries was performed prior to cluster generation and sequencing on an Illumina NovaSeq 6000 platform (Illumina, Santa Clara, CA, USA) using a single-end 1 × 75 bp format. FASTQ files underwent quality control using FastQC v0.12.1.

MicroRNA identification and quantification were performed using sRNAbench (v2022) in library mode, with the recommended settings for the library preparation kit and the “protocol=Q” option, using the Homo sapiens miRBase reference (release 22.1). MicroRNAs were retained if they had at least 3 raw reads in a number of samples equal to or greater than the size of the smaller experimental group, according to the DESeq2 guidelines (version 1.50.1; Bioconductor 3.22) Data normalization was performed in R v4.5.2 using negative binomial generalized linear models. Raw and DESeq2-normalized miRNA counts for all samples are provided in Tables S8 and S9. Differential expression analysis between samples was then performed with the DESeq2 design “~group” and with the following comparisons: “MCI vs. CTR”, “AD vs. MCI”, “AD vs. CTR” and “MCI + AD vs. CTR”. The combined MCI + AD versus CTR comparison was performed in order to identify differences shared across clinical stages that the individual comparisons were underpowered to detect. The three groups were comparable for age and sex (Table 2); these variables were therefore not included as covariates in the DESeq2 design, and the analyses presented are not adjusted for covariates.

miRNAs with a signed fold change ≥1.50 or ≤−1.50 and an adjusted *p*-value ≤ 0.05 (Benjamini–Hochberg correction) were considered significantly differentially expressed. The Benjamini–Hochberg correction was applied within each comparison and not across the four comparisons performed. Signed fold change values were obtained by converting DESeq2 log2 fold changes to linear fold changes (FC = 2^log2FC^) and transforming values below 1 as −1/FC. Data visualization included principal component analysis (PCA) and volcano plots generated with ggplot2 (version 4.4.3) and heatmaps generated with ComplexHeatmap (version 2.20.0).

### 4.5. Target Prediction and Functional Enrichment Analysis

To retrieve putative targets of differentially expressed miRNAs, the multiMiR R package (v1.24.0 and database version 2.4.0) [47] was utilized, specifically integrating interactions from two major databases of validated targets, miRTarBase [48] and TarBase [49] and two major databases of predicted targets, TargetScan [50] and miRDB [51]. To ensure high-confidence results, only the top 50% of validated and predicted targets (based on multiMiR ranking scores) were retained for downstream analysis.

The background gene universe for the enrichment tests was constructed using the complete set of targets identified from all expressed miRNAs in our dataset, applying the same top 50% filtering criteria to ensure statistical consistency.

Functional enrichment of the retrieved target sets for Gene Ontology Biological Processes (GO-BP) was conducted using the clusterProfiler R package (v4.10.1) [52]. Only pathways and functional categories reaching statistical significance after multiple-testing correction (adjusted *p*-value < 0.05) were identified as significant and considered for further interpretation. The *p*-values were adjusted using the Benjamini–Hochberg (BH) procedure. Redundant GO terms were consolidated using the simplify function to enhance clarity.

The enrichment results were visualized using dot plots that displayed the GeneRatio, count and adjusted *p*-value for the top enriched terms.

All data are publicly available in ArrayExpress under accession number E-MTAB-17063.

## 5. Conclusions

In conclusion, these findings identify miRNAs in L1CAM-enriched EVs as candidates for further investigation as minimally invasive markers in cognitive impairment. The differences observed between clinically defined groups will need to be examined in larger, independent, and biomarker-confirmed cohorts, with longitudinal follow-up, before any conclusion can be drawn about their relationship to AD pathology or their diagnostic value. Further, our functional analyses, based on database-annotated targets, point to biological processes relevant to AD pathology, including synaptic organisation and protein degradation; these associations are correlative and would require mechanistic validation before any therapeutic implications can be considered.

## Figures and Tables

**Table 1:** Differential expression of L1CAM-enriched EV-derived miRNAs across comparisons between mild cognitive impairment (MCI), Alzheimer’s disease (AD), and healthy controls (CTR).

	MCI vs. CTR			MCI + AD vs. CTR			AD vs. MCI		
miRNA	FC	*p*-Value	FDR	FC	*p*-Value	FDR	FC	*p*-Value	FDR
hsa-mir-423-5p	5.007	0.000036	**0.004**	3.072	0.002242	0.090	−4.400	0.000332	**0.034**
hsa-mir-122-5p	5.122	0.000292	**0.018**	3.070	0.00789	0.105	−5.089	0.000665	**0.034**
hsa-mir-151b	6.200	0.000647	**0.025**	3.651	0.015922	0.147	−7.366	0.000873	**0.034**
hsa-mir-16-5p	6.459	0.00883	**0.025**	3.651	0.005222	0.097	−4.475	0.008105	0.142
hsa-mir-149-3p	−5.611	0.001032	**0.025**	−2.65	0.026848	0.172	3.203	0.037801	0.279
hsa-mir-660-5p	12.506	0.003032	0.052	12.309	0.000626	**0.044**	−1.034	0.967758	0.968
hsa-mir-765	−7.069	0.00413	0.061	−5.779	0.000735	**0.044**	1.148	0.847969	0.893

Note: AD = Alzheimer’s disease, MCI = mild cognitive impairment, CTR = healthy control, FC = fold change, *p*-value = the nominal, FDR = false discovery rate adjusted for multiple testing. FDR statistically significant values are shown in bold.

**Table 2:** Demographic characteristic of the study cohorts.

	AD (n = 11)	MCI (n = 11)	CTR (n = 14)
Age at sampling, years (mean ± SD)	73.4 ± 6.6	69.9 ± 8.3	70.1 ± 8.0
M/F ratio (%M)	3/8 (27.3%)	4/7 (36.4%)	5/9 (37.5%)
Onset (mean ± SD)	66.9 ± 7.6	66.6 ± 9.0	NA
MMSE (mean ± SD)	12.8 ± 5.3	20.5 ± 8.0	28.4 ± 1.2

Note: M = male, F = female, SD = standard deviation, MMSE = mini-mental state examination, AD = Alzheimer’s disease, MCI = mild cognitive impairment, CTR = healthy controls.

## Data Availability

The data presented in this study are openly available in [ArrayExpress] at [https://www.ebi.ac.uk/biostudies/arrayexpress/studies/E-MTAB-17063?key=e15018e8-b030-4f59-bc2f-a39738a03d50 (accessed on 11 May 2026)], reference number [E-MTAB-17063].

## Supplementary Materials

The following supporting information can be downloaded at https://www.mdpi.com/article/10.3390/ijms27209301/s1.

## Abbreviations

The following abbreviations are used in this manuscript:

Aβ	amyloid-beta
AD	Alzheimer’s disease
APP	amyloid precursor protein
BH	Benjamini–Hochberg
BP	biological process
CNS	central nervous system
CSF	cerebrospinal fluid
CTR	healthy control
EV	extracellular vesicle
FDR	false discovery rate
GO	Gene Ontology
IR	insulin resistance
L1CAM	L1 cell adhesion molecules
MCI	mild cognitive impairment
miRNA	microRNAs
MMSE	mini-mental state examination
NFT	neurofibrillary tangle
NGS	Next-Generation Sequencing
NINCDS–ADRDA	National Institute of Neurological and Communicative Disorders and Stroke–Alzheimer’s Disease and Related Disorders Association
NTA	nanoparticle tracking analysis
PET	positron emission tomography
SD	standard deviation
TUBB3	β-III-tubulin
TEM	transmission electron microscopy
