# Supplementary material for: L1CAM-Enriched Extracellular Vesicle MicroRNAs in Mild Cognitive Impairment and Alzheimer’s Disease: An Exploratory Study
Source: Int J Mol Sci. 2026 Oct 20;27(20):9301. doi: 10.3390/ijms27209301 (PMC13704956; doi:10.3390/ijms27209301)
Supplement: Supplementary file 1 [file ijms-27-09301-s001.zip › Supplementary materials captions.pdf]

Supplementary file captions

Figure S1. Characterisation of the L1CAM-enriched EV fraction isolated from plasma. *All panels derive from a single pooled preparation, obtained by combining equal volumes of the final L1CAM-enriched fraction from 12 randomly selected participants (CTR n = 4, MCI n = 4, AD n = 4). (A) Nanoparticle Tracking Analysis (NTA) profile showing the particle size distribution of the pooled preparation; a video frame is also displayed. The red line represents the average size distribution resulting from five captures (videos). (B) Immunoblotting showing the presence of the EV markers ALIX and CD63 and of L1CAM and the neuron-enriched marker β-III-tubulin (TUBB3). (C) TEM image showing typical round/oval vesicle morphology; one representative field from a single grid is shown. Scale bar: 200 nm.*

Figure S2. Principal Component Analysis of miRNA expression profiles in L1CAM-enriched EVs, showing the overall variance and distribution of samples across the three cohorts*. The x-axis represents the first principal component (PC1), accounting for 23% of the total variance, while the y-axis represents the second principal component (PC2), accounting for 9% of the variance. Each data point represents a single individual, color-coded according to clinical condition: healthy controls (CTR, red), Mild Cognitive Impairment (MCI, light blue), and Alzheimer's Disease (AD, green). Shapes indicate the diagnostic group.*

Table S1. miRNA expression in the comparison between MCI vs CTR

Table S2. miRNA expression in the comparison between AD vs MCI

Table S3. miRNA expression in the comparison between AD plus MCI vs CTR

Table S4. miRNA expression in the comparison between AD vs CTR

Table S5. Gene Ontology Biological Processes targeted by up- and down-regulated miRNAs in the MCI vs CTR comparison

Table S6. Gene Ontology Biological Processes targeted by up- and down-regulated miRNAs in the AD vs MCI comparison

Table S7. Gene Ontology Biological Processes targeted by up- and down-regulated miRNAs in the AD plus MCI vs CTR comparison

Table S8. miRNAs raw count;

Table S9. miRNAs normalized count.
